# Denosumab for treating periprosthetic osteolysis; study protocol for a randomized, double-blind, placebo-controlled trial

**DOI:** 10.1186/s12891-016-1036-5

**Published:** 2016-04-23

**Authors:** Olof Sköldenberg, Agata Rysinska, Thomas Eisler, Mats Salemyr, Henrik Bodén, Olle Muren

**Affiliations:** Department of Clinical Sciences, Division of Orthopaedics, Karolinska Institutet at Danderyd Hospital, S-182 88 Danderyd, Sweden

**Keywords:** Total hip arthroplasty, Osteolysis, Denosumab, Randomized clinical trial, Outcome, Computed tomography

## Abstract

**Background:**

Wear-induced osteolysis is the main factor in reducing the longevity of total hip arthroplasty (THA). The transmembrane Receptor Activator of Nuclear Factor κ B (RANK) and its corresponding ligand RANKL is an important regulator of osteoclast activity and bone resorption and is associated with osteolysis around implant. Inhibiting RANKL with denosumab is effective in vivo in preventing osteoporosis-related fractures. In vitro, osteoclasts can be blocked in animal models of osteolysis. We hypothesize that denosumab is effective in reducing wear-induced osteolysis around uncemented acetabular implants in THA.

**Methods/design:**

A randomized, double-blind, placebo-controlled trial will be conducted. We will include 110 patients, 40–85 years of age, with a known osteolytic lesion around an uncemented acetabular component ≥7 years after the primary operation. The patients will be randomized in a 1:1 ratio to subcutaneous injections of 60 mg denosumab or placebo for a total of 6 doses with start on day one and every 6 months with last treatment at 30 months. The primary endpoint will be the change in volume of the osteolytic lesion at 3 years measured with three-dimensional computed tomography (3D-CT). Secondary endpoints include functional outcome scores, change in bone mineral density of the lumbar spine, serological markers of bone turnover and adverse events.

**Discussion:**

In vitro results of both bisphosphonates and RANKL inhibitors have been promising, showing reduced osteolysis with treatment. This is, to our knowledge, the first clinical trial testing the efficacy of denosumab in reducing wear-induced osteolysis. The study is an academic, phase II trial from an independent center and is designed to demonstrate efficacy in reducing volume of osteolytic lesions around a total hip arthroplasty.

**Trial registration:**

ClinicalTrials.gov (NCT02299817) 2014-11-20

## Background

Total hip arthroplasty (THA) is one of the most cost-effective [1] and quality of life restoring [2] surgical procedure in orthopaedics and more than 2 million patients undergo THA worldwide annually. Although THA generally leads to remarkably good outcomes, more than 100 000 patients each year have to undergo a risky and costly revision surgery due to aseptic loosening caused by osteolysis. This cell mediated inflammatory response to wear debris from the artificial joint is the major factor in reducing the longevity of a THA [3–5] The risk of failure is highest in younger males with a 30 % risk for revision surgery within 10 years [6, 7]. Despite continual changes in surgical technique and implant design, the revision THA burden (defined as the percentage of revision THA cases as a function of all THA cases) has not decreased over time and is currently around 10 % in Sweden and 17 % in the US [8, 9].

Osteolytic lesions around well-fixed orthopaedic implants are notoriously difficult to detect and are, 7–14 years after surgery, present in 10-70 % of hips [10, 11]. They are in almost all cases asymptomatic and can only be detected with a reasonably good sensitivity and specificity using computed tomography (CT) or magnetic resonance tomography [12]. The lesions typically occur more than 5 year after surgery [11] and, when extensive, undermine the bony fixation of the implant thereby leading to loosening of the artificial joint. The extent of the revision surgery and subsequent result for the individual patient is strongly correlated to the size of the osteolytic lesion. There is little data on the development and progression of osteolysis around hip implants and there are few studies where osteolytic lesions have been systematically followed over a number of years using CT or MRI [13, 14].

The transmembrane Receptor Activator of Nuclear Factor κ B (RANK) and its corresponding ligand RANKL is an important regulator of osteoclast activity and bone resorption and is associated with osteolysis around implants [15–17]. The wear particles from polyethylene in the artificial joint induce the over-expression of RANK, inflammatory cytokines interleukin (IL)-6, IL-8, interferon-β-inducible protein (IP)-10, monocyte chemoattractant protein (MCP)-1, monokine induced by interferon-β (MIG) in the microenvironment around the implant [18]. This susceptibility to develop osteolysis has been shown to vary between individuals [19, 20].

Bisphosphonates have been found to be effective in reducing disuse bone atrophy (a.k.a. “stress-shielding”) around orthopaedic implants but have not been effective in preventing progress of osteolytic lesions [21, 22]. Cathepsin K is a protease that is responsible for the degradation of bone matrix by osteoclasts. Inhibitors of cathepsin K are in development for treatment of osteoporosis and have recently been show to reduce fracture risk in patients with osteoporosis [23]. The mechanism for Cathepsin K inhibitors could potentially also be used for treatment of osteolysis; however, there are currently no drugs available for use.

A MedLine search on the Mesh terms Osteolysis, Hip Arthroplasty, Bisphosphonates, RANKL/RANK and Medical treatment fails to find any studies on this subject and in effect, there is as of yet no medical treatment available.

Recently denosumab was found to be effective in preventing osteoporosis related fractures in post-menopausal women [24] by blocking RANKL and thereby inhibiting the development and activity of osteoclast. In a recently published animal model of prosthetic loosening, targeting osteoclast recruitment via RANKL inhibition was found to be effective in targeting osteoclast [25]. Denosumab has however, to the best of our knowledge, not been used to try to prevent the progression of osteolysis and aseptic loosening in THA.

The problem with osteolysis and subsequent loosening around implants is equally pronounced for titanium hemispherical acetabular components with polyethylene liners as well as when the polyethylene is fixed to the host bone with bone cement. In the U.S. and Europe titanium hemispherical acetabular components with polyethylene liners are the most common acetabular components and this study will therefore aim to treat patients with these types of implants. If denosumab is effective in treating osteolytic lesions it would have an immense impact since revision surgery is costly [26] and the results after surgery are uncertain.

We hypothesize that denosumab is effective in reducing wear-induced osteolysis around uncemented acetabular implants in THA.

## Methods/design

### Study design

A randomized, double-blind, placebo-controlled trial will be conducted. Patients will be randomized in a 1:1 ratio to placebo or denosumab using concealed envelopes. A randomly assigned batch size of 4 to 10 (in increments of 2; thus 4, 6, 8, or 10) will be used. Osteolytic lesion volume (<10 cm^3^/≥10 cm^3^) at screening and physical activity according to Johnston [27] (<4-low activity level/≥4-high activity level) are clear risk factors for progression of volume size and will therefore be used as stratification to ensure that these are evenly distributed between the two groups [13, 27, 28]. The patients and all staff and investigators will be blinded to treatment. The study will be carried out from 2015 to 2021 at the Orthopaedic Department of Danderyd Hospital in collaboration with the Karolinska Institute. The Ethics Committee of the Karolinska Institute and the Swedish Medical Products Agency approved the study. The trial is initiated, designed, and performed as an academic investigation and registered at ClinicalTrials.gov (NCT02299817). The guidelines of the CONSORT Statement will be followed [29].

### Study population

**Fig. 1:**
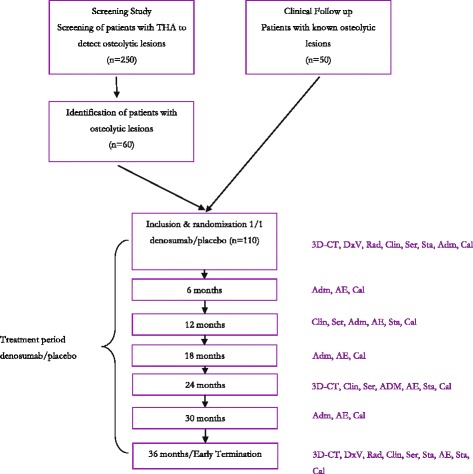
3D-CT = Three-dimensional volumetric computed tomography, DxV = DXA scan vertebrae L1-L4, Rad = Anterioposterior and lateral radiographs, Clin = clinical follow-up including Harris hip score, EQ-5D, Activity scores and Pain Numerical Rating Scale, Ser = serological markers of bone turnover markers, Sta = standard blood test, Adm = Administration of IMP/placebo, AE = AE/SAE assessment, Cal = S-Calcium

Patients with titanium press-fit acetabular components with polyethylene liners will be screened for osteolytic lesions using three-dimensional computed tomography (3D-CT). The patients had their primary surgery more than 7 years before the screening and were all operated because of osteoarthritis of the hip. This time period is sufficient for osteolytic lesions to appear. In this patient group we expect that approximately 30 % of screened patients will have an osteolytic lesion. These patients with screening-detected lesions as well as patients with previously known osteolytic lesions will be eligible for inclusion in the study (Fig. 1). We will include asymptomatic patients, aged 40–85 years, with a primary THA performed due to osteoarthritis or congenital dysplasia of the hip ≥ 7 years before inclusion and who have a osteolytic lesion of at least 4 cm^3^ and at most 40 cm^3^ around an uncemented acetabular component with a polyethylene liner. Exclusion criteria includes pain from the hip (VAS ≥3), any surgery of the hip after index operation, ever use of bisphosphonates and inflammatory arthritis. A detailed inclusion and exclusion list is presented in Table 1. The inclusion period is planned from 2015 to 2016. End of study (EOS) will be at the last included patient’s last visit.

**Table 1:** Detailed inclusion and exclusion criteria

Inclusion criteria	Exclusion criteria
Age 40–85 years	For women of childbearing potential: Subject refuses to use 1 highly effective method of contraception (contraceptive pill, intra uterine contraceptive device) for the duration of the study and for 10 months after the last dose of study medication.
Short Portable Mental Status Questionnaire (SPMSQ) also named Pfeiffertest ≥7	For males with a partner of childbearing potential: Subject refuses to use 1 highly effective method of contraception for the duration of the study and for 10 months after the last dose of study medication.
Male and females	For males with a partner who is pregnant: Subject refuses to use a condom for the duration of the study and for 10 months after the last dose of study medication.
The primary THA performed between 7 to 20 years before inclusion.	Pain in the operated hip (because the presence of hip pain in combination with an osteolytic lesion is an indication for revision surgery). VAS >3
The primary THA performed due to osteoarthritis or congenital dysplasia of the hip.	Previous revision surgery of the hip i.e. exchange of any inplant after the primary surgery
Uncemented cup fixation	Inflammatory arthritis
Baseline osteolytic lesion of at least 4 cm^3^ and at most 40 cm^3^around an uncemented acetabular component with a polyethylene liner.	Previous participation in clinical trials with denosumab or administration of commercial denosumab (Prolia™ or Xgeva™)
Participant is willing and able to follow study protocol and has provided informed consent prior to any study specific procedures.	Currently enrolled in or has not yet completed at least 1 month since ending other investigational device or drug trial (s), or subject is receiving other investigational agent (s).
	Treatment with any intravenous bisphosphonate, fluoride (except for dental treatment) or strontium ranelate within 5 years prior to inclusion.
	Treatment with any oral bisphosphonate within 1 year prior to inclusion.
	Treatment with cortisol or cytostatic drugs within 6 months prior to inclusion.
	Administration of any of the following treatments 3 months prior to screening:
	For women of childbearing potential: Subject refuses to use 1 highly effective method of contraception (contraceptive pill, intra uterine contraceptive device) for the duration of the study and for 10 months after the last dose of study medication.
	For males with a partner of childbearing potential: Subject refuses to use 1 highly effective method of contraception for the duration of the study and for 10 months after the last dose of study medication.
	For males with a partner who is pregnant: Subject refuses to use a condom for the duration of the study and for 10 months after the last dose of study medication.
	Pain in the operated hip (because the presence of hip pain in combination with an osteolytic lesion is an indication for revision surgery). VAS >3
	Previous revision surgery of the hip i.e. exchange of any inplant after the primary surgery
	Inflammatory arthritis
	Previous participation in clinical trials with denosumab or administration of commercial denosumab (Prolia™ or Xgeva™)
	Currently enrolled in or has not yet completed at least 1 month since ending other investigational device or drug trial (s), or subject is receiving other investigational agent (s).
	Treatment with any intravenous bisphosphonate, fluoride (except for dental treatment) or strontium ranelate within 5 years prior to inclusion.
	Treatment with any oral bisphosphonate within 1 year prior to inclusion.
	Treatment with cortisol or cytostatic drugs within 6 months prior to inclusion.
	Administration of any of the following treatments 3 months prior to screening:
	*Anabolic steroids or testosterone*
	*Glucocorticosteroids (≥5 mg prednisone equivalent per day for more than 10 days or a total cumulative dose of ≥ 50 mg)*
	*Calcitonin*
	*Calcitriol or vitamin D derivatives [vitamin D contained in supplements or multivitamins is allowed]*
	*Other bone active drugs including anti-convulsives (except benzodiazepines) and heparin*
	*Chronic systemic ketoconazole, ACTH (adrenocorticotrophic hormone), cinacalcet, aluminum, lithium, protease inhibitors, methotrexate, gonadotropin-releasing hormone agonists.*
	*Androgen deprivation therapy*
	Hypocalcaemia.
	Bone metabolic disorders (such as OI, PHPT, Paget)
	History of osteonecrosis of the jaw and/or recent tooth extraction or dental surgery; or planned invasive dental proceedures during the study
	Serum 25-OH D <20 ng/ml
	Significant malabsorption including Celiac Disease, Short Bowel Syndrome, Crohn’s Disease, Previous Gastric Bypass.
	Active cancer and/or malignancy in last 5 years (except cervical carcinoma in situ or basal cell carcinoma)
	History of solid organ or bone marrow transplant.
	Hypersensitivity to any components of study drug.
	Intolerance to calcium supplements.
	Pregnancy and/or currently lactating.
	Significantly impaired renal function as determined by a derived glomerular filtration rate (GFR) using Cockcroft Gault formula of ≤ 30 mL/min/1.73 m2
	Elevated transaminases ≥ 2.0 × upper limit of normal (ULN); Elevated total bilirubin (TBL) > 1.5 × ULN.
	Any condition or illness (acute, chronic, or history), which in the opinion of the Investigator might interfere with the evaluation of efficacy and safety during the study or may otherwise compromise the safety of the subject.
	Hypocalcaemia.
	Bone metabolic disorders (such as OI, PHPT, Paget)
	History of osteonecrosis of the jaw and/or recent tooth extraction or dental surgery; or planned invasive dental proceedures during the study
	Serum 25-OH D <20 ng/ml
	Significant malabsorption including Celiac Disease, Short Bowel Syndrome, Crohn’s Disease, Previous Gastric Bypass.
	Active cancer and/or malignancy in last 5 years (except cervical carcinoma in situ or basal cell carcinoma)
	History of solid organ or bone marrow transplant.
	Hypersensitivity to any components of study drug.
	Intolerance to calcium supplements.
	Pregnancy and/or currently lactating.
	Significantly impaired renal function as determined by a derived glomerular filtration rate (GFR) using Cockcroft Gault formula of ≤ 30 mL/min/1.73 m2
	Elevated transaminases ≥ 2.0 × upper limit of normal (ULN); Elevated total bilirubin (TBL) > 1.5 × ULN.
	Any condition or illness (acute, chronic, or history), which in the opinion of the Investigator might interfere with the evaluation of efficacy and safety during the study or may otherwise compromise the safety of the subject.

### Treatments

Half of the patients will receive a 1 ml subcutaneous injection of denosumab 60 mg on day one and every 6 months with last treatment at 30 months (day 1, 6 mos., 12 mos., 18 mos., 24 mos., 30 mos.), total 6 times. The other half will receive placebo injections as above. The selected dose 60 mg is established in clinical routine for treatment of osteoporosis. The syringes are to be stored at 2-8 °C in a locked, temperature controlled refrigerator. All used syringes must be saved and stored in a cabinet to be checked by the monitor. Study supplies will be documented by completion of drug accountability forms. They will include details of the quantities of IMP/placebo received, administered, stored and dispatched or destroyed. Calcium and Vitamin D (500 mg + 400 IE) twice daily will be given from day 1 to 3 years to all patients. All personnel involved in the study will be blinded. Code envelopes will be stored at the investigator’s office to be used for emergency reasons. In case of a suspected serious adverse drug reaction (SADR) during the study, the PI will determine if unblinding of the study drug is necessary. This decision will be taken by the PI within 24 h of his knowledge of the event. If the PI, for some reason, is unavailable, one of the co-investigators will handle the decision of unblinding.

### When and how to withdraw subjects from the trial treatment

Patients with serious adverse events (SAE’s) with a direct impact on the periprosthetic region studied will be withdrawn from treatment. Examples of such SAE’s are periprosthetic fractures after accidents, deep infections with revision surgery or any kind of surgery in the hip studied. Should a subject request or decide to withdraw from the study, all efforts will be made to complete and report the observations as thoroughly as possible up to the date of withdrawal. For withdrawn subjects the last post-baseline observation will be carried forward. If the subject withdraws consent or is excluded from the study a 3D-CT will be performed and serological bone turnover markers will be collected (SCTx and P1NP). If implants are to be revised during the study, a CT will be done before surgery and serological bone turnover markers will be collected (SCTx and P1NP). In a case of withdrawal of full consent, the subject will be followed according to the routine standard follow-up of THA patients at our institution, including regular clinical examinations and radiographic follow-up visit at the operating surgeon every 2–3 years.

### Endpoints and follow-up

The primary endpoint variable will be the change in volume of the osteolytic lesion over 3 years (measured with 3D-CT in cm^3^):

$$ Efficac{y}_{3 years}= Volum{e}_{3 years}- Volum{e}_{baseline}. $$

Secondary endpoints include change in volume of the osteolytic lesion over 2 years, percentage change of the lesion over the study period, clinical outcome scores and bone turnover measurements (Table 2). Depending on the outcome parameters, measurements will take place at screening, 6, 12, 18, 24, 30 and 36 months (Fig. 1).

**Table 2:** Secondary endpoints

No.	Outcome measurement	Follow-up time
1	Baseline data including height, weight, medical history, physical examination	Screening
2.	Hip outcome scores; Harris hip score [36], WOMAC [35]	Screening, 12, 24, 36 months
3.	Pain Numerical Rating Scale (PNRS), Activity Scores [27] and Health-related quality of life (EQ-5D) [37]	Screening, 12, 24, 36 months
4^a^	Percent change from baseline in BMD in vertebrae L1-L4 measured with dual-energy x-ray absorptiometry (DXA)	Screening, 36 months
5.	Correlation between change in bone turnover markers and progression of osteolysis. Serum C-terminal telopeptide of type I collagen (SCTx) [42] and procollagen type 1 amino-terminal propeptide (P1NP) [43].	Screening, 12, 24, 36 months
6.	Correlation between change in serum concentrate values for RANKL and Osteoprogesterin (OPG) and progression of osteolysis^b^.	Screening, 24,36 months
7.	Occurrence of adverse events	6, 12, 18, 24, 30, 36 months
8.	Radiological analysis plain x-ray	Screening, 36 months

^a^Previous studies on denosumab have focused on patients with osteoporosis or other metabolic bone disease and it is to be expected that the patients in this trial will have a normal bone mass

^b^Serum levels of RANKL and OPG will be quantified by ELISA with commercially available matched antibodies [44, 45]

### Osteolysis assessment

We will use a high-resolution three dimensional computed tomography (3D-CT) at inclusion to detect and measure the volume of the osteolysis according to Howie et al. [13, 30]. The scan will be repeated at 2 and 3 years. Osteolysis will be defined as a demarcated nonlinear osteolytic lesion >3 mm. The measurements will be performed by a technician otherwise not involved in the study and blinded to treatment and who is trained in quantitative CT analysis. 3D-CT has been shown to have an 80 % sensitivity and a 100 % specificity in detecting osteolytic lesions [12] around uncemented acetabular components. Once detected the volume of the lesion can be measured with an error of mean (SD) 7.1 % ±24.1 % (0.3 ± 1.1 cm^3^) [12].

### Radiological and bone densitometric assessment

Plain x-rays of the hip and femur will be taken at baseline and at 3 years to measure wear of the polyethylene. The two-dimensional (2-D) linear head penetration rate will be measure measured from the postoperative examination and inclusion examination using the software Hip Analysis Suite™ (University of Chicago, Chicago, Illinois, USA) version 8.0.4.1 [31] This method uses conventional AP radiographs and the software uses image analysis techniques, determination of bone landmarks and edge detection algorithms to determine the 2-D penetration value change in the position of the femoral head centre with respect to the acetabular component centre. The radiographs will also be examined at 3 years for signs of atypical femoral fractures. There are three reported cases of atypical femoral fractures after denosumab treatment but all of those had, prior to denosumab treatment, been treated with long-term bisphosphonate treatment [32–34]. Bone mineral density (BMD) of the lumbar spine (vertebrae L1 through L4) will be measured at inclusion and at 3 years using dual x-ray absorptiometry (DXA) (DPX-L; Lunar, Madison, Wisconsin, USA) The BMD will be categorized according to the World Health Organization (WHO) classification for osteoporosis.

### Clinical safety assessments and withdrawal from study

Adverse events (AEs) are defined as any untoward medical occurrence in a patient or clinical investigation subject administered a pharmaceutical product and that does not necessarily have a causal relationship with this treatment. Adverse events include serious adverse events (SAEs), adverse drug reactions (ADRs), serious adverse drug reactions (SADRs) and will be assessed throughout the study period for all patients (Table 3). In case of an adverse event, or serious adverse events, treatment and follow-up will be performed according to clinical routine. The investigator will ensure that all events observed by the investigator or reported by the subject that occur throughout the trial period, starting from the time when a subject has signed the informed consent through to 30 days after the last dose of IP or the EOS (excluding the long-term follow-up period) which ever is longer, are reported using the applicable CRF and properly captured in the patients’ medical records. The investigator will record and grade all adverse events according to Table 3. The investigator will assess whether the adverse event is possibly related to the IP. This relationship is indicated by a response to the question: “Is there a reasonable possibility that the event may be related to a study activity”? The investigator will review laboratory test results and determining whether an abnormal value in a trial subject represents a change from the subject’s baseline values. Abnormal laboratory findings without clinical significance (based on the investigator’s judgment) will not be recorded as adverse events. A patient may also voluntarily withdraw from treatment due to what he or she perceives as an intolerable adverse event. If either of these situations arises, the patient should be strongly encouraged to undergo an end-of-study assessment and be under medical supervision until symptoms cease or the condition becomes stable.

**Table 3:** Definition of safety assessments

No.	Type	Definition
1.	Adverse event (AE)	An adverse event is defined in the International Conference on Harmonization (ICH) Guideline for Good Clinical Practice as “any untoward medical occurrence in a patient or clinical investigation subject administered a pharmaceutical product and that does not necessarily have a causal relationship with this treatment.” (ICH E6:1.2). The investigator is responsible for reviewing laboratory test results and determining whether an abnormal value in an individual study subject represents a change from values before the study. In general, abnormal laboratory findings without clinical significance (based on the investigator’s judgment) should not be recorded as adverse events; however, laboratory value changes requiring therapy or adjustment in prior therapy are considered adverse events.
2.	Serious adverse event (SAE)	A serious adverse event (SAE) is defined as an adverse event that meets at least 1 of the following criteria: a) fatal, b) life threatening (places the subject at immediate risk of death), c) requires in-patient hospitalization or prolongation of existing hospitalization, d) results in persistent or significant disability/incapacity or congenital anomaly/birth defect e) other significant medical hazard. A hospitalization meeting the regulatory definition for “serious” is any inpatient hospital admission that includes a minimum of an overnight stay in a health care facility. Any adverse event that does not meet one of the definitions of serious (e.g., emergency room visit, outpatient surgery, or requires urgent investigation) may be considered by the investigator to meet the “other significant medical hazard” criterion for classification as a serious adverse event. Examples include allergic bronchospasm, convulsions, and blood dyscrasias.
3.	Adverse drug reaction (ADR)	All untoward and unintended responses to a medicinal product related to any dose administered. The phrase “responses to a medicinal product” means that a causal relationship between the medicinal product and the adverse event is at least a reasonable possibility, i.e. the relationship cannot be ruled out.
4.	Serious adverse drug reaction (SADR)	A serious ADR (SADR) is an ADR that meets the definition of SAE
5.	AE attributes	The investigator will assign the following adverse event attributes:
		Adverse event diagnosis or syndrome (s), if known (if not known, signs or symptoms)
		Dates of onset and resolution
		Severity
		Assessment of relatedness to IP
		Action taken.
6	AE grading	The following adverse events severity grading scale used in the trial.
		MILD: Aware of sign or symptom, but easily tolerated
		MODERATE: Discomfort enough to cause interference with usual activity
		SEVERE: Incapacitating with inability to work or do usual activity
		LIFE-THREATENING: Refers to an event in which the patient was, in the view of the investigator, at risk of death at the time of event.
		FATAL

### Functional outcome

The Harris hip score (HHS) and WOMAC score will be used to assess patient-reported functional hip status, and physical activity [35, 36]. Health-related quality of life will be assessed by the EQ-5D (EuroQoL), which uses five dimensions: mobility, self-care, usual activity, pain/discomfort and anxiety/depression [37]. Each dimension is divided into three levels as follows: 1, no problems; 2, some problems; and 3, extreme problems. This generates 243 different ‘health states’ and the EQ-5D index score. Pain from the hip will be recorded using the Pain Numeric Rating Scale (PNRS), which is an 11-point (0 to 10) scale, in which 0 denotes no pain and 10 unbearable pain [38].

### Data quality assurance

The study progress and study conduct will be monitored before, during and after the study to ensure that ICH-GCP, regulatory requirements, and all aspects of the protocol are followed. The medical records and other documents will be reviewed for verification of agreement with data on the Case Report Forms (CRFs). The subject has a right for a protection against invasion of privacy. All study data will be collected and managed in a digital CRF using REDCap electronic data capture tools hosted at Karolinska Institutet [39]. REDCap (Research Electronic Data Capture) is a secure, web-based application designed to support data capture for research studies, providing: 1) an intuitive interface for validated data entry; 2) audit trails for tracking data manipulation and export procedures; 3) automated export procedures for seamless data downloads to common statistical packages; and 4) procedures for importing data from external sources. In this study, each subject will receive a unique identification number, which will be linked to the CRF. The data will then be blinded correspondingly in all data analyses. However, the study monitor, auditor, representative from any Regulatory Authority, as well as the appropriate Ethical Committee are permitted to review the subject’s primary medical records including laboratory test result reports, ECG reports, admission and discharge summaries, AE and SAE reports occurring during the study.

### Sample size

#### Assumptions for sample size

In a pilot study using 3D-CT Schwarz et al. [40] identified 19 patients with osteolytic lesions around an uncemented acetabular cup used in THA. After 1 year the volume of the lesions had increased with mean (SD) 3.19 (3.67) cm^3^. Howie et al. [14] studied the natural progression of osteolytic lesions after THA with 3D-CT. He scanned 30 patients with a known osteolytic lesion 15 months (range, 12–27) after the initial scan and found that 16 (53 %) of the lesions had increased in volume. The lesions most likely to increase in size was ≥10 cm^3^ at the initial scan. The median volume increase was 3 cm^3^ during the 15 months studied. Based on the work by Schwarz [40] and Howie [14] and thereby assuming a 3 cm^3^ increase annually and a 3 year study period would indicate that we are looking for a mean increase of 9 cm^3^ with a SD of 8 cm^3^ [14]. The SD is estimated by dividing Howie et al.’s range of lesion size divided by 4 as suggested by Hozo et al. [41]. For denosumab we assume that it will reduce the progression of osteolysis about 50 % compared to placebo. Patients treated with denosumab would therefore have a mean increase of 4.5 cm^3^ (0.5 × 9 cm^3^) after 3 years.

#### Sample size calculation

A two-tailed superiority sample size calculation for the primary endpoint variable change in osteolytic volume after 3 years, assuming a progression of volume of 9 cm^3^ for the placebo and 4.5 cm^3^ for the denosumab group and with a SD of 8 cm^3^ in both groups and a *p*-value of 0.05 means 50 subjects in each group for a 80 % power. We will include 55 patients in each group to allow for loss to follow-up and loss of data. We therefore need to identify 110 patients with osteolytic lesions and include them in the study.

### Statistics

The analyses will be performed on the basis of the intention-to-treat principle, and all patients who receive at least one injection of either denosumab or placebo will be included in the final analysis. We will use the unpaired Student’s t-test and Levene’s test for comparison of change in osteolysis volume at 2 and 3 years. Descriptive statistics (means and standard deviations) will be used to describe the patient characteristics and outcome variables at the measurement points. An analysis of covariance (ANCOVA) of the primary endpoint including terms for treatment group, stratification factors and with age and sex as confounders will be performed. ANCOVA will also be used for numeric secondary outcome variables such as progression of osteolysis at 2 years, change in vertebrae 1–4 BMD and the contralateral hip and biochemical markers of bone turnover. For hip-specific outcomes score (Harris hip score, WOMAC) and health-related quality of life (EQ-5D) we will use non-parametric tests. For subjects that withdrawn from the study before year 3 the data from the last observation will be carried forward (imputed). Safety data will be summarized with descriptive statistics.

## Discussion

This is the first study on denosumab and osteolysis. We will include patients with asymptomatically osteolytic lesions. These lesions are, based on the current literature, highly likely to progress over the years and lead to massive osteolysis and require revision surgery. Revision surgery is significantly more risky for the individual patient than a primary THA. The risk of dislocation and deep periprosthetic joint infection is, for example, 4–10 times more common after revision arthroplasty than after a primary THA. The clinical outcome regarding hip function is also poorer after revision surgery. Although our proposed main outcome is volume of osteolysis and not a clinical endpoint, the study is designed as a proof-of-concept and the results, if positive, could be inferred to larger patient groups.

The risks of denosumab treatments are low, the side-effects reported are benign. There are two reported cases of atypical femoral fractures after denosumab treatment but all of those had, prior to denosumab treatment, been treated with long-term bisphosphonate treatment. Any previous bisphosphonate treatment is an exclusion criteria in the current trial. In summary, we believe the benefit of the trial outweighs the risk for the individual patient.

### Ethics approval and consent to participate

The Ethics Committee of the Karolinska Institute and the Swedish Medical Products Agency approved the study. Individual consent will be obtained from each patient.

### Consent to publish

Not applicable.

### Availability of data and materials

De-identified data will be available from the authors institution by request.

### Study status

The study is ongoing and recruiting patients.

### Related articles

No related articles for this study has been published.

## Abbreviations

3D-CT: three-dimensional computed tomography
ADR: Adverse drug reaction
AE: adverse event
ANCOVA: analysis of co-variance
BMD: Bone mineral density
CONSORT: consolidated standards of reporting trials
CRF: Case report form
CT: computed tomography
DXA: dual x-ray absorptiometry
ECG: electrocardiogram
EOS: End of study
EQ-5D: a standardised instrument for use as a measure of health outcome
EuroQoL: European quality of life scale
HHS: Harris hip score
ICH-GCP: international conference on harmonisation-good clinical practice
IMP: investigational medicinal product
MCP: monocyte chemoattractant protein
MIG: monokine induced by interferon-β
MRI: magnetic resonance imaging
P1NP: Procollagen type 1 amino-terminal propeptide
PI: principal investigator
PNRS: Pain Numeric Rating Scale
RANK: receptor activator of nuclear factor kappa B
RANKL: receptor activator of nuclear factor kappa B ligand
SADR: Serious drug reaction
SAE: Serious adverse event
SCTx: Serum C-terminal telopeptide of type I collagen
THA: total hip arthroplasty
WOMAC: Western Ontario and McMaster Universities arthritis index

## References

[1] Learmonth ID, Young C, Rorabeck C (2007). The operation of the century: total hip replacement. Lancet.

[2] Ng CY, Ballantyne JA, Brenkel IJ (2007). Quality of life and functional outcome after primary total hip replacement. A five-year follow-up. J Bone Joint Surg (Br).

[3] Dumbleton JH, Manley MT, Edidin AA (2002). A literature review of the association between wear rate and osteolysis in total hip arthroplasty. J Arthroplasty.

[4] Harris WH (1995). The problem is osteolysis. Clin Orthop Relat Res.

[5] Harris WH (2001). Wear and periprosthetic osteolysis: the problem. Clin Orthop Relat Res.

[6] Ahnfelt L, Herberts P, Malchau H, Andersson GB (1990). Prognosis of total hip replacement. A Swedish multicenter study of 4,664 revisions. Acta Orthop Scand.

[7] Malchau H, Herberts P, Ahnfelt L (1993). Prognosis of total hip replacement in Sweden. Follow-up of 92,675 operations performed 1978–1990. Acta Orthop Scand.

[8] Kurtz S, Mowat F, Ong K, Chan N, Lau E, Halpern M (2005). Prevalence of primary and revision total hip and knee arthroplasty in the United States from 1990 through 2002. J Bone Joint Surg Am.

[9] Bozic KJ, Kurtz SM, Lau E, Ong K, Vail TP, Berry DJ (2009). The epidemiology of revision total hip arthroplasty in the United States. J Bone Joint Surg Am.

[10] Isaac DL, Forder J, Skyrme AD, James SE (2007). The Biomet Bi-Metric total hip arthroplasty and universal acetabular cup: high polyethylene failure rate in the medium term. J Arthroplasty.

[11] Marshall A, Ries MD, Paprosky W (2008). How prevalent are implant wear and osteolysis, and how has the scope of osteolysis changed since 2000?. J Am Acad Orthop Surg.

[12] Leung S, Naudie D, Kitamura N, Walde T, Engh CA (2005). Computed tomography in the assessment of periacetabular osteolysis. J Bone Joint Surg Am.

[13] Howie DW, Neale SD, Martin W, Costi K, Kane T, Stamenkov R, Findlay DM (2012). Progression of Periacetabular Osteolytic Lesions. J Bone Joint Surg Am.

[14] Howie DW, Neale SD, Stamenkov R, McGee MA, Taylor DJ, Findlay DM (2007). Progression of acetabular periprosthetic osteolytic lesions measured with computed tomography. J Bone Joint Surg Am.

[15] Andersson MK (2005). Biological aspects on synovial fluid mediated aseptic prosthesis loosening.

[16] Andersson MK, Lundberg P, Ohlin A, Perry MJ, Lie A, Stark A, Lerner UH (2007). Effects on osteoclast and osteoblast activities in cultured mouse calvarial bones by synovial fluids from patients with a loose joint prosthesis and from osteoarthritis patients. Arthritis Res Ther.

[17] Lerner UH, Ohlin A (1993). Tumor necrosis factors alpha and beta can stimulate bone resorption in cultured mouse calvariae by a prostaglandin-independent mechanism. J Bone Miner Res.

[18] Wang CT, Lin YT, Chiang BL, Lee SS, Hou SM (2010). Over-expression of receptor activator of nuclear factor-kappaB ligand (RANKL), inflammatory cytokines, and chemokines in periprosthetic osteolysis of loosened total hip arthroplasty. Biomaterials.

[19] Gordon A, Greenfield EM, Eastell R, Kiss-Toth E, Wilkinson JM (2010). Individual susceptibility to periprosthetic osteolysis is associated with altered patterns of innate immune gene expression in response to pro-inflammatory stimuli. J Orthop Res.

[20] Gallo J, Raska M, Mrazek F, Petrek M (2008). Bone remodeling, particle disease and individual susceptibility to periprosthetic osteolysis. Physiol Res.

[21] Talmo CT, Shanbhag AS, Rubash HE (2006). Nonsurgical management of osteolysis: challenges and opportunities. Clin Orthop Relat Res.

[22] Bhandari M, Bajammal S, Guyatt GH, Griffith L, Busse JW, Schunemann H, Einhorn TA (2005). Effect of bisphosphonates on periprosthetic bone mineral density after total joint arthroplasty. A meta-analysis. J Bone Joint Surg Am.

[23] Makras P, Delaroudis S, Anastasilakis AD (2015). Novel therapies for osteoporosis. Metabolism.

[24] Cummings SR, San Martin J, McClung MR, Siris ES, Eastell R, Reid IR, Delmas P, Zoog HB, Austin M, Wang A (2009). Denosumab for prevention of fractures in postmenopausal women with osteoporosis. N Engl J Med.

[25] Aspenberg P, Agholme F, Magnusson P, Fahlgren A (2011). Targeting RANKL for reduction of bone loss around unstable implants: OPG-Fc compared to alendronate in a model for mechanically induced loosening. Bone.

[26] Ong KL, Mowat FS, Chan N, Lau E, Halpern MT, Kurtz SM (2006). Economic burden of revision hip and knee arthroplasty in Medicare enrollees. Clin Orthop Relat Res.

[27] Johnston RC, Fitzgerald RH, Harris WH, Poss R, Muller ME, Sledge CB (1990). Clinical and radiographic evaluation of total hip replacement. A standard system of terminology for reporting results. J Bone Joint Surg Am.

[28] Lubbeke A, Garavaglia G, Barea C, Stern R, Peter R, Hoffmeyer P (2011). Influence of patient activity on femoral osteolysis at five and ten years following hybrid total hip replacement. J Bone Joint Surg (Br).

[29] Schulz KF, Altman DG, Moher D (2010). CONSORT 2010 statement: updated guidelines for reporting parallel group randomised trials. BMJ.

[30] Howie DW, Neale SD, Martin W, Costi K, Kane T, Stamenkov R, Findlay DM (2012). Progression of periacetabular osteolytic lesions. J Bone Joint Surg Am.

[31] Martell JM, Berdia S (1997). Determination of polyethylene wear in total hip replacements with use of digital radiographs. J Bone Joint Surg Am.

[32] Thompson RN, Armstrong CL, Heyburn G (2014). Bilateral atypical femoral fractures in a patient prescribed denosumab - a case report. Bone.

[33] Schilcher J, Aspenberg P (2014). Atypical fracture of the femur in a patient using denosumab--a case report. Acta Orthop.

[34] Drampalos E, Skarpas G, Barbounakis N, Michos I (2014). Atypical femoral fractures bilaterally in a patient receiving denosumab. Acta Orthop.

[35] Bellamy N (1989). Pain assessment in osteoarthritis: experience with the WOMAC osteoarthritis index. Semin Arthritis Rheum.

[36] Harris WH (1969). Traumatic arthritis of the hip after dislocation and acetabular fractures: treatment by mold arthroplasty. An end-result study using a new method of result evaluation. J Bone Joint Surg Am.

[37] Rabin R, de Charro F (2001). EQ-5D: a measure of health status from the EuroQol Group. Ann Med.

[38] Downie WW, Leatham PA, Rhind VM, Wright V, Branco JA, Anderson JA (1978). Studies with pain rating scales. Ann Rheum Dis.

[39] Harris PA, Taylor R, Thielke R, Payne J, Gonzalez N, Conde JG (2009). Research electronic data capture (REDCap)--a metadata-driven methodology and workflow process for providing translational research informatics support. J Biomed Inform.

[40] Schwarz EM, Campbell D, Totterman S, Boyd A, O’Keefe RJ, Looney RJ (2003). Use of volumetric computerized tomography as a primary outcome measure to evaluate drug efficacy in the prevention of peri-prosthetic osteolysis: a 1-year clinical pilot of etanercept vs. placebo. J Orthop Res.

[41] Hozo SP, Djulbegovic B, Hozo I (2005). Estimating the mean and variance from the median, range, and the size of a sample. BMC Med Res Methodol.

[42] Vasikaran S, Eastell R, Bruyere O, Foldes AJ, Garnero P, Griesmacher A, McClung M, Morris HA, Silverman S, Trenti T (2011). Markers of bone turnover for the prediction of fracture risk and monitoring of osteoporosis treatment: a need for international reference standards. Osteoporos Int.

[43] Samoszuk M, Leuther M, Hoyle N (2008). Role of serum P1NP measurement for monitoring treatment response in osteoporosis. Biomark Med.

[44] Aukrust P, Muller F, Lien E, Nordoy I, Liabakk NB, Kvale D, Espevik T, Froland SS (1999). Tumor necrosis factor (TNF) system levels in human immunodeficiency virus-infected patients during highly active antiretroviral therapy: persistent TNF activation is associated with virologic and immunologic treatment failure. J Infect Dis.

[45] Ueland T, Bollerslev J, Godang K, Muller F, Froland SS, Aukrust P (2001). Increased serum osteoprotegerin in disorders characterized by persistent immune activation or glucocorticoid excess--possible role in bone homeostasis. Eur J Endocrinol.

